# A Physiologically Based Pharmacokinetic Model to Predict Systemic Ondansetron Concentration in Liver Cirrhosis Patients

**DOI:** 10.3390/ph16121693

**Published:** 2023-12-06

**Authors:** Faleh Alqahtani, Abdullah H. Alruwaili, Mohammed S. Alasmari, Sultan A. Almazroa, Khaled S. Alsuhaibani, Muhammad F. Rasool, Abdulkarim F. Alruwaili, Sary Alsanea

**Affiliations:** 1 Department of Pharmacology and Toxicology, College of Pharmacy, King Saud University, Riyadh 11451, Saudi Arabia; afaleh@ksu.edu.sa (F.A.); 442105382@student.ksu.edu.sa (A.H.A.); sultanmazroa@hotmail.com (S.A.A.); khaledssuh@gmail.com (K.S.A.); 2 Department of Pharmacy Practice, Faculty of Pharmacy, Bahauddin Zakariya University, Multan 60800, Pakistan; fawadrasool@bzu.edu.pk; 3 Clinical Pharmacy Unit, Department of Pharmaceutical Services, Dallah Hospital, Riyadh 12381, Saudi Arabia; a_alruwaili@dallah-hospital.com

**Keywords:** ondansetron, physiologically based pharmacokinetics, liver cirrhosis, nausea, vomiting, simulation

## Abstract

Introduction: Ondansetron is a drug that is routinely prescribed for the management of nausea and vomiting associated with cancer, radiation therapy, and surgical operations. It is mainly metabolized in the liver, and it might accumulate in patients with hepatic impairment and lead to unwanted adverse events. Methods: A physiologically based pharmacokinetic (PBPK) model was developed to predict the exposure of ondansetron in healthy and liver cirrhosis populations. The population-based PBPK simulator PK-Sim was utilized for simulating ondansetron exposure in healthy and liver cirrhosis populations. Results: The developed model successfully described the pharmacokinetics of ondansetron in healthy and liver cirrhosis populations. The predicted area under the curve, maximum systemic concentration, and clearance were within the allowed twofold range. The exposure of ondansetron in the population of Child–Pugh class C has doubled in comparison to Child–Pugh class A. The dose has to be adjusted for liver cirrhosis patients to ensure comparable exposure to a healthy population. Conclusion: In this study, the developed PBPK model has described the pharmacokinetics of ondansetron successfully. The PBPK model has been successfully evaluated to be used as a tool for dose adjustments in liver cirrhosis patients.

## 1. Introduction

Ondansetron was approved by the United States Food and Drug Administration (FDA) to be used medically as a prototype for a new class of antiemetics in 1991 [1]. It works as a serotonin 5-HT3 (5-hydroxytryptamine3) receptor antagonist [2]. It is a carbazole molecule containing nitrogen rings and carbon that resemble serotonin structurally, enabling it to bind to the 5-HT3 receptor and exert its clinical effects [3]. The 5-HT3-type serotonin receptors exist both centrally and peripherally, in the chemoreceptor trigger zone (CTZ) and on vagal nerve terminals, respectively. Ondansetron inhibits the CTZ region in the brain that controls the nausea reflex by blocking serotonin [4]. Ondansetron is indicated for the management of nausea and vomiting that are associated with cancer, radiation therapy, and surgical operations. In addition, it falls into pregnancy risk category B, so it is considered the most commonly recommended for the prophylaxis of hyperemesis gravidarum, a severe form of sickness during pregnancy [5]. There are some side effects that are associated with the use of ondansetron, including headache, constipation, diarrhea, asthenia, drowsiness, and cardiovascular and central nervous system side effects [6]. Moreover, significant adverse reactions have been reported, including QT prolongation interval, torsade de pointes, and bradycardia. Therefore, the medication should be administered cautiously to individuals exhibiting a prolonged QT interval or those at risk of drug accumulation, such as those with compromised hepatic or renal function [7].

Following oral administration, ondansetron undergoes rapid absorption, becoming detectable in plasma within 30 min. It exhibits an absolute bioavailability of around 60–70%, with 30–40% of the drug being eliminated during the initial pass through metabolism. The bound fraction to plasma protein has been estimated to be 70–76%, and the volume of distribution (Vd) and elimination half-life (t_1/2_) are 1.8 L/kg and 3.8 ± 1 h, respectively [8,9]. Ondansetron is widely metabolized by the liver, with only around 5% of the dose excreted unchanged from the kidney. It is a substrate for multiple cytochrome P450 enzymes, including 1A1, 1A2, 2D6, and the 3A subfamily, with CYP2D6 playing less of a role than others [10]. The oxidation process is the major metabolic pathway through which several metabolites are produced, including 8-hydroxy-ondansetron, 7-hydroxy-ondansetron, and 6-hydroxy-ondansetron, in ratios of 40%, <20%, and <5%, respectively [8]. The total body clearance of ondansetron ranges from 600 to 700 mL/min, and renal clearance is as low as 20 mL/min, accounting for less than 5% of the total clearance [9].

Recently, there has been an increase in the utilization of mathematical models during drug development processes. The concept of using mathematical models to mechanistically describe pharmacokinetic processes was first introduced in 1937 by Teorell [11]. Physiologically based pharmacokinetic (PBPK) models are mechanistic models that are developed by incorporating the physicochemical properties of drugs with previous knowledge of the physiology to predict pharmacokinetic parameters. This approach allows for the incorporation of tissue composition and blood flow to the organs for predicting drug pharmacokinetics (PKs). A PBPK model for the whole body has a clear illustration of the tissues that are linked to the pharmacokinetic processes, including absorption, distribution, excretion, and metabolism (ADME). In comparison to other pharmacokinetic approaches, the main characteristic feature of PBPK models is the availability of a complete structural representation of an organism’s anatomy and physiology [12]. These models can be used for ‘what if scenarios’ to predict drug exposure in untested medical conditions with subsequent dosing recommendations. These models have been successfully used to predict the PKs of different drugs in special populations (geriatric, pediatric, and obese) and disease populations (renal, hepatic, and heart failure) [13,14,15,16]. Ondansetron is used to treat a variety of medical conditions, and it is prescribed for a wide range of populations that might have special considerations, such as hepatic dysfunction. Since a high fraction of the administered dose is metabolized in the liver, any change in the blood flow to the liver or in the intrinsic clearance can have an impact on ondansetron’s PKs, suggesting adjustments in administered doses for avoidance. Therefore, applying the concept of PBPK modeling will be useful for predicting ondansetron PK in patients with various degrees of liver impairment [17].

The pathophysiological changes in specific diseases, such as liver cirrhosis, can be integrated into the model of PBPK for PK prediction of drugs according to the severity of liver dysfunction [18,19]. Even though there are some published PBPK models for ondansetron, they have not been applied to predict drug exposure in hepatic cirrhosis patients with various degrees of disease severity. The goal of this current work is to develop a PBPK model comprising the whole body by utilizing a systematic model-building approach for predictions of ondansetron PKs in patients with various degrees of liver impairment. The developed model will be used to suggest dosing regimens according to the functional status of the liver cirrhosis population.

## 2. Results

### 2.1. Healthy PBPK Model Development and Evaluation

**Figure 1:**
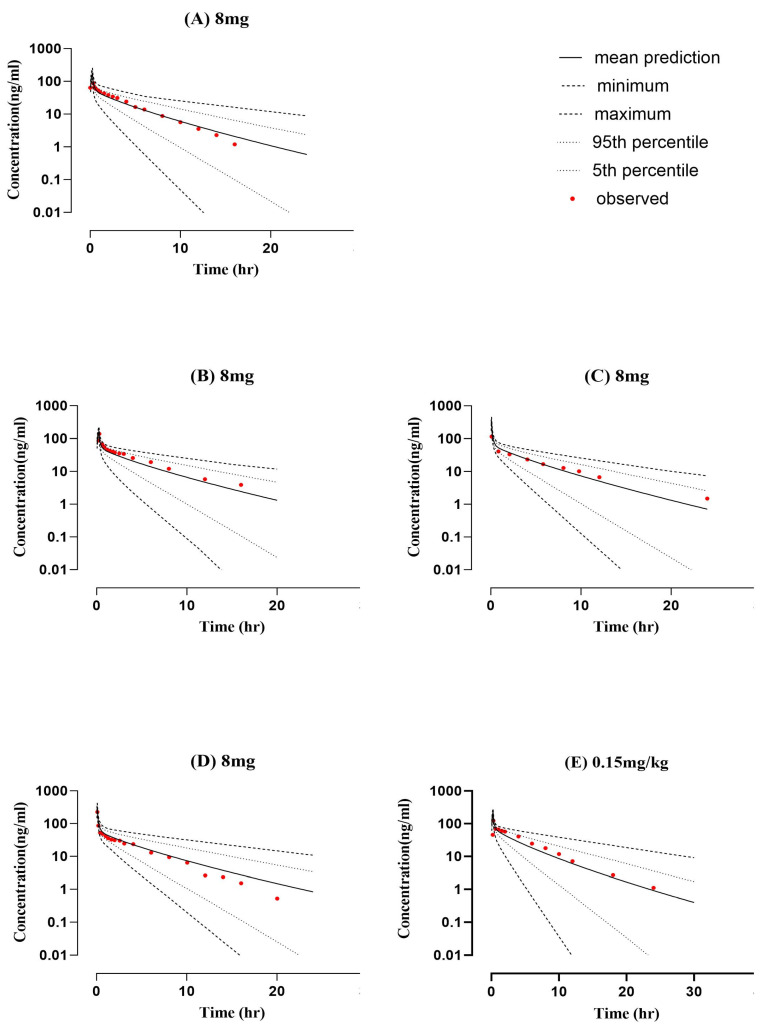
Comparison of predicted and observed systemic concentration vs. time profile in healthy subjects after intravenous application of ondansetron (8 mg dose). (**A**) 8 mg [20], (**B**) 8 mg [21], (**C**) 8 mg [22], (**D**) 8 mg [23], and (**E**) 8 mg [24].

**Figure 2:**
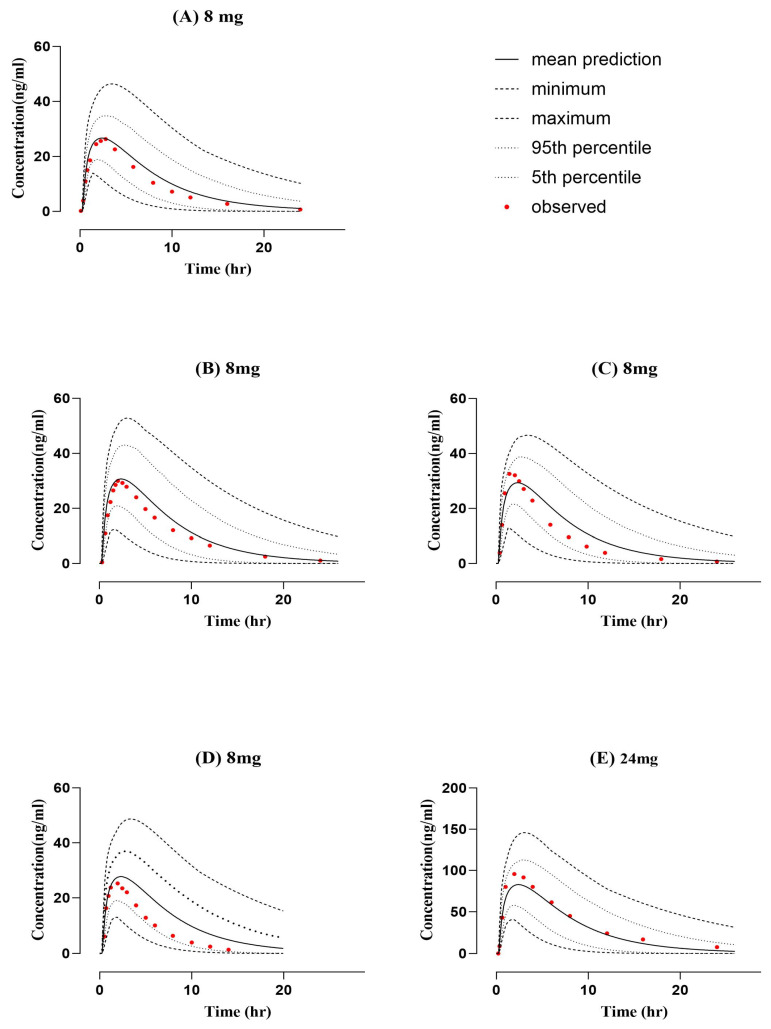
Comparison of predicted and observed systemic concentration vs. time profile in a healthy population after oral administration of ondansetron (8 mg dose). (**A**) 8 mg [25], (**B**) 8 mg [26], (**C**) 8 mg [24], (**D**) 8 mg [20], and (**E**) 24 mg [27].

As shown in Figure 1 and Figure 2, the performance of the developed PBPK models for a healthy population was acceptable, given that all the systemic concentration versus time profiles of observed data fall within the prediction interval. The visual verification showed that the simulated model has been successfully interpreted with observed PK data after intravenous and oral application in healthy individuals according to comparison with the clinical PK data, 10–90th percentile, min., and max. simulated concentration curves. Also, Robs/Rpre ratios have been determined for all pharmacokinetic parameters, such as Cmax, AUC, and CL, to confirm the model’s accuracy. The ratio of Robs/pre mean for AUC time 0 infusion after intravenous administration was 0.98 and ranged between 0.82 and 1.17, whereas after oral administration it was 0.87 and ranged between 0.68 and 1.04. Cmax and CL mean values after IV administration were 0.77 ng/mL and 1.08 mL/min/kg, whereas after oral administration they were 1.07 ng/mL and 1.23 mL/min/kg, respectively. The calculated Cmax and CL ratios can be seen in Table 1. All PK parameters were within the acceptable range of twofold error. Furthermore, Table 2 describes the average fold error and root mean square error values for prediction of all doses (intravenous and oral) used for the ondansetron-PBPK model development and evaluation.

### 2.2. PBPK Model Development and Evaluation for Cirrhotic Patient Population

**Figure 3:**
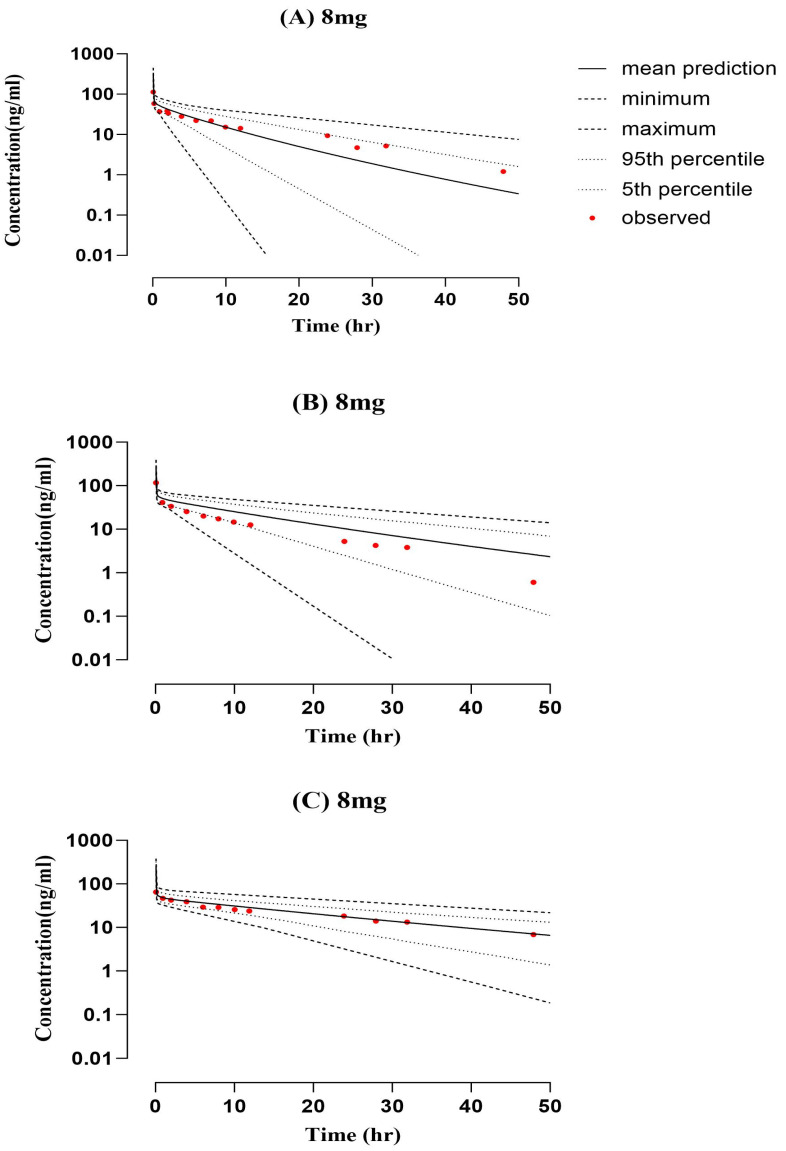
Comparison of predicted and observed systemic concentration vs. time in hepatic failure subjects after 8 mg ondansetron intravenous administration. (**A**) 8 mg [22], (**B**) 8 mg [22], and (**C**) 8 mg [22].

The cirrhosis model was extrapolated from the healthy model following IV administration of an 8 mg dose (Figure 3). A comparison of observed and predicted systemic concentration versus time profiles was conducted to evaluate the accuracy of the developed acceptable twofold error range. The observed/predicted ratios of AUC0-Inf after IV 8 mg administration in CP-A, CP-B, and CP-C were 1.33, 0.80, and 1.14 ng/mL, respectively. The values and ratios of AUCs, Cmax, and CL can be seen in Table 3. The accumulation of the drug in this population was described precisely using the model with respect to the Child–Pugh classification system for liver disease severity.

**Figure 4:**
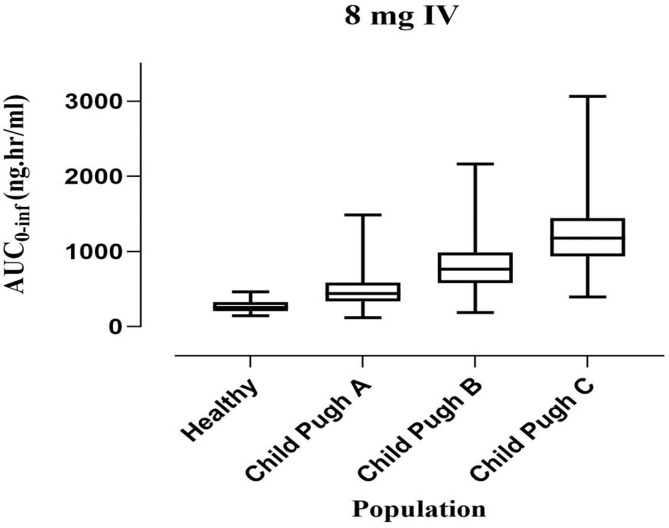
Comparing the AUC (5–95th percentile) after an IV 8 mg ondansetron dose in healthy, CP-A (mild), CP-B (moderate), and CP-C (severe) liver cirrhosis populations.

For dose optimization, we simulated the plasma concentration with a gradual decrease in the dose to have a comparable PK parameter to a healthy population. We used box-whisker plots to show the effect of dosing optimization on the PK parameter. However, the median with 95% confidence interval (CI) for AUC0-inf in healthy (IV 8 mg) is 312.4 (271.06–372.41), which significantly increased to 476.46 (104.55–1552.99) in CP-A, 801.90 (171.74–2185.99) in CP-B, and 1208.94 (369.41–3067.14) in CP-C (Figure 4).

**Figure 5:**
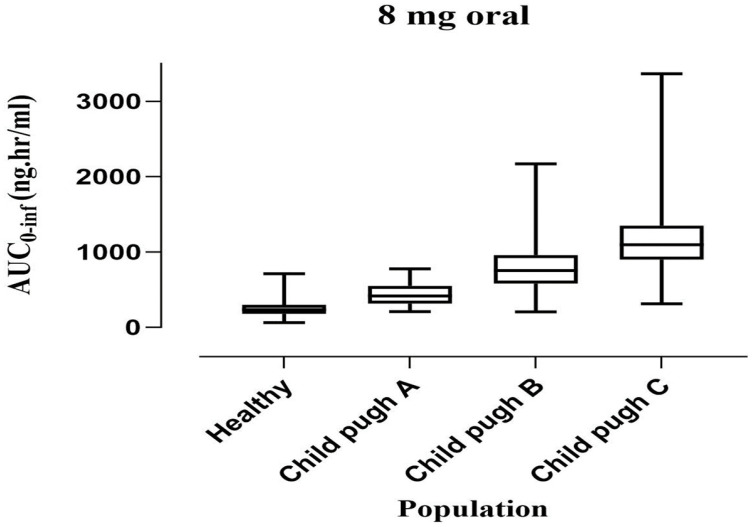
Comparing AUC (5–95th percentile) after an oral 8 mg ondansetron dose in healthy, Child–Pugh A (mild), B (moderate), and C (severe) hepatic impairment populations.

The model was extrapolated from healthy to liver cirrhosis after ondansetron 8 mg oral administration. The box-whisker plots were employed for dosage optimization, and the AUC was compared for liver cirrhosis. The median was 260.1 (246.96–280.70) with a 95% CI for AUC0-inf in the healthy population, which increased to 444.61 (80.22–1575.57) in CP-A, 773.84 (188.39–2088.88) in CP-B, and 1114.82 (296.27–3423.62) in CP-C. The visual inspection of time profiles suggested that the developed cirrhotic population PBPK model was reasonable (Figure 3). Each PK parameter’s resultant Robs/Rpre ratio was within the 2-fold error range. The box plots that illustrate these variations are shown in Figure 5.

The reported model has successfully explained the PKs of ondansetron after IV and oral application in healthy and diseased individuals. As a result of these changes in AUC, which are associated with liver cirrhosis disease, dose adjustments can be achieved to use ondansetron in populations with liver cirrhosis. Ultimately, these findings will help adjust doses in liver cirrhosis patients.

## 3. Discussion

In this investigation, PBPK models for ondansetron were developed following oral and intravenous doses in both healthy and diseased populations (liver cirrhosis). This involved employing a systematic approach to construct a comprehensive physiologically based PKs model. Following the precedent set by previously published models, the initial assessments were conducted in the healthy population before extending the evaluations to the population with the disease. Ondansetron is metabolized mainly in the liver into 8-hydroxy-ondansetron (40%), 7-hydroxy-ondansetron (<20%), and 6-hydroxy-ondansetron (<5%) via cytochrome P450 enzymes through CYP3A4 and CYP1A2, whereas CYP2D6 plays less of a role in metabolism. Noticeably, the clearance of ondansetron is principally through the hepatic metabolism, at 95% compared to less than 5% via the kidney. According to impaired organ function, these drug-metabolizing CYP enzymes are altered in response to hepatic disease status [8]. The renal clearance of ondansetron is about 20 mL/min of 600–700 mL/min total plasma clearance [9]. It is now clear that patients suffering from liver cirrhosis are more susceptible to developing complications when taking drugs that are mainly cleared through the liver.

In this current study, we sought to develop and validate a PBPK model based on the reported physiochemical properties and concentration profiles of the compound in the literature for oral and IV ondansetron administration in healthy subjects by using the in silico technique. Based on our results, the predicted and observed values demonstrate that they complied with each other, as supported by the mean AUC0-inf value of 312.4 ng.hr/mL vs. 306.2 ng.hr/mL after intravenous dose. Moreover, following oral administration, the mean observed AUC0-inf value of 227.5 ng.hr/mL was in line with the predicted AUC0-t (260.1 ng.hr/mL).

Ondansetron may be used as long-term therapy for several medical conditions, especially neoplastic drug-induced nausea and vomiting. There is a significant alteration in the oral bioavailability of ondansetron in cancer patients due to their first past metabolism, which averaged 85 to 87% compared to only 50–70% in healthy volunteers in earlier studies [28]. Moreover, two additional studies reported a higher blood concentration of ondansetron through liver impairment [22,29]. PK parameters of ondansetron, such as AUC, Cmax, t1/2, bioavailability, and volume distribution, were greater in patients with liver disease, whereas CL was lower. Eventually, the time between doses should be prolonged. A single daily dose is sufficient [9]. So, there is the importance of identifying ondansetron–PK properties. In extrapolation of the model to liver cirrhosis, the disease-specific pathophysiological changes obtained from reported literature were reductions such as blood flow to the organs, glomerular filtration rate, liver volume, hematocrit, and plasma protein concentrations, which will ultimately increase the risk of developing irreversible complications [18,19]. Moreover, the CP classification is used to determine the degree of liver impairment. It is essential to determine how these changes may affect the pharmacodynamics and PKs of drugs, potentially resulting in adverse reactions or therapeutic failure. The AUC0-inf was found to be significantly increased compared to the control by 34.43%, 61.04%, and 74.16% in liver cirrhosis populations, mild (CP-A), moderate (CP-B), and severe (CP-C), respectively, after intravenous application. Whereas, following the oral administration, the AUC0-inf was increased by 41.50%, 66.40%, and 76.70% in CP-A, CP-B, and CP-C, respectively, which suggested that dosage adjustment may be required in hepatic impairment.

The strength of this current study is that, so far, no PBPK model for liver disease populations has been published on ondansetron. Previously, there were studies published related to ondansetron PBPK models for pregnant and pediatric populations.

This study has a few limitations. Some included studies for model evaluation did not highlight gender proportion or equal gender distribution. The ondansetron clinical PK data containing concentration versus time profiles were limited to healthy populations, and only one study was available for IV administration in liver cirrhosis populations; therefore, the model-predicted data in oral administration for disease populations cannot be verified. Moreover, in order to validate the presented model, more clinical PK data are required, and this is a potential limitation of the presented work.

## 4. Materials and Methods

### 4.1. Clinical Pharmacokinetic Data

A comprehensive literature search was conducted using Google Scholar and NCBI-PubMed databases to identify the relevant clinical PK studies with reported systemic ondansetron concentrations in order to be used for model development and evaluation purposes. If the concentration versus time points were presented graphically, we used GetData Graph Digitizer^®^ (version.2.26.0) (available from Software.informer.com, data accessed on 15 May 2023) to extract the experimental data from the plasma concentration versus time profiles. A total of 10 studies (5 for each intravenous and oral administration) in healthy populations and one study in the liver cirrhosis patient population were eligible to be used for the development of the PBPK models. The severity of liver cirrhosis in the study was assessed according to Child–Pugh (CP) liver cirrhosis scores and classified into CP-A for mild, CP-B for moderate, and CP-C for severe [22]. All the PK studies that were used for developing and verifying the models are summarized in Table 4.

### 4.2. PBPK Modeling Software

The population-based simulator PK-Sim^®^ (version 11) was employed to develop PBPK models for ondansetron in healthy adults and cirrhotic populations. This software is part of Bayer Technology Services GmbH’s Systems Biology Software Suite (Leverkusen, Germany) for PBPK modeling and simulation [30]. A free version of the PK-Sim program is available at (http://www.open-systems-pharmacology.org, data accessed on 15 May 2023) for all users, which is part of open systems pharmacology.

### 4.3. Development of Building Blocks

The model was parameterized using drug-specific, biological system-specific, and clinical trial-related data collected through the literature search. Then all the data were incorporated into the building blocks. In order to predict drug exposure more accurately, certain parameters were fitted after parameter identification. All plasma concentration datapoint profiles were scanned and imported from the included studies using Excel sheets for the noncompartmental analysis (NCA). The PBPK model parameters are summarized in Table 5.

### 4.4. Modeling Strategy

**Figure 6:**
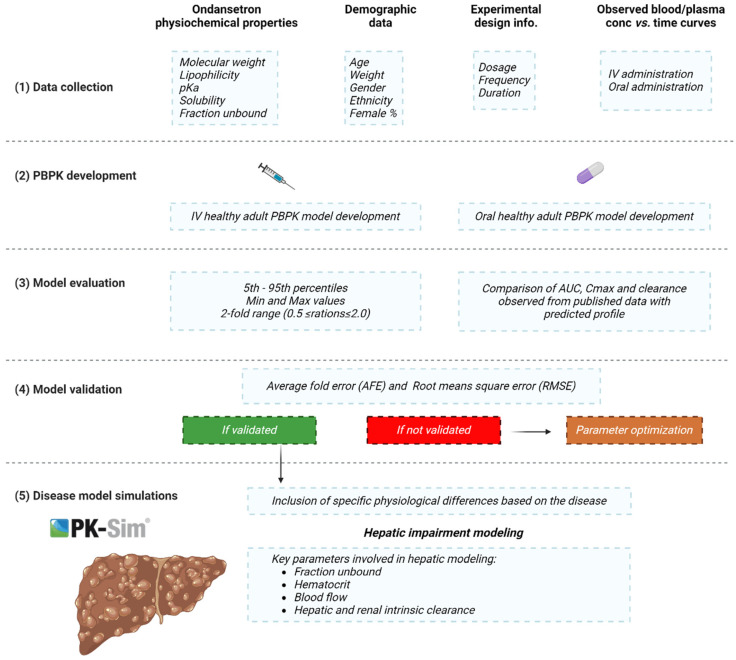
Overall workflow of ondansetron PBPK model development in cirrhotic and healthy populations. The figure was created with BioRender^®^.

The standard protocol for PBPK model development and evaluation has been followed as described in the literature. Since absorption is a complex process that involves multiple compartments, drug disposition was modeled first using PK data after IV administration. Since the liver is the major eliminating organ for ondansetron, with CYP1A2 and CYP3A4 enzymes being the most important contributors to the metabolic process, the intrinsic clearance of these enzymes was used as a major determinant of the elimination phase. In addition, renal clearance was used for the remaining unexplained elimination process, as explained in Table 5. The distribution between compartments was determined using the molecular weight and the fraction unbound. This process is explained by using the differential equations provided by the program. A virtual human population consisting of 1000 subjects was created for every simulation based on the reference clinical study in terms of proportion of females, frequency, dose, age, weight, and ethnicity. After the development and evaluation of the distribution and elimination models, the absorption phase was modeled and evaluated subsequently with respect to the observed data. A general scheme for the modeling strategy is depicted in Figure 6.

### 4.5. Model Parametrization

As illustrated in Table 2, ondansetron is a basic drug with a pKa value of 7.40, a molecular weight of 293.4 g/mol, and a log P of 2.25. The lipophilicity of ondansetron reported in the literature ranged from 2.1 to 2.4 [31,32]. Using ondansetron’s lipophilicity and molecular weight, the specific intestinal permeability was calculated as 2.24 × 10^−5^ cm/min, which was then incorporated into the developed PBPK model. For the estimation of tissue plasma partition coefficients, the Poulin and Theil model was applied. Cellular permeability was estimated using the Pk-Sim standard model [36]. To describe the elimination process, the intrinsic clearance (CLint) values of the CYP enzymes responsible for the metabolism of ondansetron were recalculated using the well-stirred liver model. Firstly, ondansetron is extensively cleared by hepatic metabolism, so CYP enzymes played the main role in elimination. The fractions metabolized by CYP3A4 and CYP1A2 of ondansetron were estimated previously [37,38]. The major CYP enzymes that are involved in the metabolism of ondansetron are CYP1A2 and CYP3A4, and the specific clearance values of CYP enzymes obtained from the literature were 0.13 for CYP1A2 and 0.02 for CYP3A4. To achieve these values, the input values were manually optimized to 0.21 and 0.03 L/min, respectively, as intrinsic clearance. Finally, a value of 0.13 mL/min/kg was incorporated into the model, which represents the minor role that the kidney plays in the elimination process.

### 4.6. Model Structure in Cirrhotic Population

There are different reported pathophysiological changes in populations who have liver cirrhosis disease. These pathophysiological changes indicate the severity of the disease. The Child–Pugh (CP) classification aids in quantifying these alterations as the disease progresses over time. Depending on these pathophysiological changes in hematocrit, blood flow, plasma protein binding factor, GFR, and liver volume, the cirrhotic PBPK model was developed as shown in Table 3 [18,19,39]. The study that was selected for ondansetron–cirrhosis PBPK model verification has mentioned a mean plasma concentration versus time curve for cirrhotic populations with various degrees of severity. The total number of participants was 19 (6 were CP-A, 6 were CP-B, and 7 were CP-C). All the CP class model predictions were visually verified by comparing the observed data with the predicted data. After that, the ondansetron–cirrhosis model was developed. Therefore, predictions for each CP class were carried out for the model verification process (Table 6).

After successful evaluation of the developed drug–disease ondansetron PBPK model, the mean predicted values after IV application were compared with the observed datasets (Table 6). Because of the increase in AUC, a reduction in dose was suggested based on a comparison of AUC between both healthy and cirrhosis populations (CP-A–C). Box-whisker plots were used to graphically represent the outcomes of dose adjustments.

### 4.7. Model Appraisal and Verification

The PBPK-developed models were developed using visual verification and comparison of predicted PK parameters with observed clinical data. The comparison of all models was carried out by comparing the predicted arithmetic mean, the 5–95th percentile, and the minimum and maximum drug plasma concentration versus time profile curves with the mean plasma concentration versus time curves of observed clinical data. Different PK parameters of the observed and predicted data were calculated using the (Microsoft365 Version 2019) Excel add-in PK-Solver^®^ by noncompartmental analysis (NCA) [40]. Then, the ratio of observed to predicted values (Robs/Rpre) was calculated to compare the various PK parameters—the mean area under the plasma concentration versus time curve from zero to infinity (AUC0-inf), maximum plasma drug concentration (Cmax), and plasma drug clearance (CL/F)—with a 95% confidence interval (CI) as shown in Table 4. As previously published, model verification was considered reasonable if the ratio of predicted to observed data was within a predefined twofold range (0.5 ≤ ratios ≤ 2.0) [41,42]. To further validate the developed PBPK mode, the average fold error (AFE) and root mean square error (RMSE) were determined for each parameter [42,43]. To suggest drug doses for cirrhosis patients (CP-A–C), the box-whisker plots were performed. For this purpose, ondansetron’s AUC0-inf., Cmax, and CL/F in healthy and diseased populations (CP-A–C) were determined. To calculate Robs/Rpre, AFE, and RMSE, the Equations (1)–(4) are given below:

(1) $$R=\frac{Observed\;value\;of\;PK\;parameter}{Predicted\;value\;of\;PK\;parameter}$$

(2) $$Fold-error=\frac{Observed\;values\;of\;parameter}{Predicted\;values\;of\;paramete}$$

(3) $$AFE=10\frac{\sum \log\left(fold\;error\right)}{N}$$

(4) $$RMSE=\sqrt{\frac{\sum_{1}^{N}{\left(observed\;PK\;parameter\;value-predicted\;PK\;parameter\;value\right)}^{2}}{N}}$$

## 5. Conclusions

Ondansetron’s PKs in healthy and cirrhotic populations has been successfully described by the developed PBPK model following oral and intravenous dosing. The assessed PBPK ondansetron disease model can have many implications for optimizing and predicting drug dosage in patients with liver cirrhosis at various disease severity stages.

## Figures and Tables

**Table 1:** The ratio of Robs/pre for PK parameters in a healthy population after ondansetron application (IV and oral).

PK Parameters (Unit)									
Dose, Reference	AUC0–∞ (ng/mL·h)			Cmax (ng/mL)			CL (mL/min/kg)		
	Obs.	Pred.	Obs./Pred Ratio	Obs.	Pred.	Obs./Pred Ratio	Obs.	Pred.	Obs./Pred Ratio
IV administration									
8-mg [20]	246.5	271.06	0.91	102.5	170.69	0.60	7.41	6.73	1.10
8 mg [22]	279	324.87	0.86	161	344.15	0.47	6.54	5.70	1.15
8 mg [23]	257	315.18	0.82	343	315.95	1.09	7.10	5.70	1.25
8 mg [21]	313	278.54	1.12	159	166.97	0.95	6.7	6.52	1.03
0.15 mg/kg [24]	435.46	372.41	1.17	170	225.86	0.75	5.81	6.76	0.86
Oral administration									
24 mg [27]	805.9	777.09	1.04	94.6	82.81	1.14	8.64	7.02	1.23
8 mg [26]	244.496	280.70	0.87	36.113	30.69	1.18	8.06	6.52	1.24
8 mg [25]	246.1	247.22	1.00	26.3	26.68	0.99	7.42	7.31	1.01
8 mg [24]	209.27	266.78	0.78	32.57	29.34	1.11	8.9	7.47	1.19
8-mg [20]	168.8	246.96	0.68	26.4	27.73	0.95	10.8	7.28	1.48

Obs, observed; Pred, predicted; IV, intravenous; AUC0–∞, area under the plasma concentration vs. time curve (time 0–infinity); Cmax, max. systemic drug concentration; CL, plasma drug clearance.

**Table 2:** Error values (average fold error) and (root mean square error) for PK parameters in a healthy population after IV and oral ondansetron administration.

Parameters	AFE	RMSE
Intravenous		
AUC	0.98	47.44
Cmax	0.77	91.77
CL	1.08	0.91
Oral		
AUC	0.87	50.90
Cmax	1.07	6.01
CL	1.23	1.94

AFE, average fold error; AUC, area under plasma concentration vs. time curve from zero to infinity; Cmax, maximum plasma drug concentration; CL, plasma drug clearance; RMSE, root mean square error.

**Table 3:** The ratio of Robs/pre for PK parameters in liver cirrhosis patients after ondansetron administration (IV).

PK Parameters (Unit)									
Dose, Reference	AUC0–∞ (ng/mL·h)			Cmax (ng/mL)			CL (mL/min/kg)		
	Obs.	Pred.	Obs./Pred Ratio	Obs.	Pred.	Obs./Pred Ratio	Obs.	Pred.	Obs./Pred Ratio
IV Administration									
(8 mg) Child–Pugh-A [22]	633	476.46	1.33	113.7	323.39	0.35	2.89	3.81	0.76
(8 mg) Child–Pugh-B [22]	641	801.90	0.80	174.6	274.22	0.64	2.84	2.28	1.25
(8 mg) Child–Pugh-C [22]	1383	1208.94	1.14	149.5	251.84	0.60	1.32	1.51	0.87

Obs, observed; Pred, predicted; IV, intravenous; AUC0–∞, area under the systemic concentration vs. time curve (time 0–infinity); Cmax, max. systemic drug concentration; CL, plasma drug clearance.

**Table 4:** Clinical studies used in ondansetron PBPK model development and evaluation.

Study	Dose	Infusion Time	n	Female [n]	Mean Age [Years] ± SD	Mean w.t [kg] ± SD	Population
Intravenous application in healthy population							
[20]	8 mg	15 min	32	0	18–40	58.3–95.8	Healthy
[22]	8 mg	5 min	6	2	19–23	N/A	Healthy
[23]	8 mg	5 min	6	2	32–43	50–80	Healthy
[21]	8 mg	15 min	6	0	19–35	55.5–90.5	Healthy
[24]	0.15 mg/kg	15 min	11	5	31 ± 7	66.1 ± 8.5	Healthy
Oral application in healthy population							
[27]	24 mg	PO	12	6	N/A	N/A	Healthy
[26]	8 mg	PO	22	11	18–41	49–94	Healthy
[25]	8 mg	PO	24	0	19–39	60–90	Healthy
[24]	8 mg	PO	11	5	31 ± 7	66.1 ± 8.5	Healthy
[20]	8 mg	PO	32	0	(18–40)	58.3–95.8	Healthy
Intravenous application in disease population							
[30]	8 mg	5 min	19	8	(20–69)	N/A	Disease

N/A: not available; PO: oral administration.

**Table 5:** Ondansetron PBPK model input parameters.

Parameter	Input Value	Reference
Physicochemical parameters		
Molecular weight (g/mol)	293.4	Pubchem
Lipophilicity (log units)	2.25	[31,32]
Plasma protein binding	Albumin	[8]
Solubility(mg/L)	0.36	Drugbank
pKa(base)	7.40	[25]
Absorption		
Specific intestinal permeability (cm/min)	2.24 × 10^−5^	Pk-Sim calculated
Distribution		
Specific organ permeability (cm/min)	8.02 × 10^−3^	Pk-Sim calculated
Fraction unbound (Fu)%	27	[33]
Partition coefficient model	Poulin and Theil	Pk-Sim
Cellular permeability model	Pk-Sim standard	Pk-Sim
Metabolism		
Intrinsic clearance CYP1A2 (L/min)	0.21	[34]
Intrinsic clearance CYP3A4 (L/min)	0.03	
Excretion		
Renal clearance (mL/min/kg)	0.13	[8,35]

**Table 6:** Physiological changes associated with liver cirrhosis.

Parameters		Control (PK Sim)	Child–Pugh Score		
			CP-A	CP-B	CP-C
Functional liver mass ^b^		2.38	0.69	0.55	0.28
Hepatic enzymes fraction (cyp) pmol/mg.	3A4 ^a,b^	4.32	0.589 ^a^	0.4 ^b^	0.4 ^b^
	1A2 ^a^	1.8	0.63 ^a^	0.26 ^a^	0.12 ^a^
Albumin conc (g/L). ^c^		1	0.84 ± 0.15	0.69 ± 0.15	0.53 ± 0.15
Haematocrit Value (%) ^b^		0.47	0.39	0.37	0.35
Blood Flow ^b^	Portal (mL/min)	1.21	0.4	0.36	0.04
	Hepatic arterial (mL/min)	17.94	1.3	2.3	3.4
	Other organs (mL/min)	-	1.75	2.25	2.75
	Renal (mL/min)	302.71	0.88	0.65	0.48
GFR (mL/min) ^a^		116	0.7	0.58	0.55

^a^ fractions of control values extracted from [18], ^b^ fractions of control values extracted from [19], ^c^ fractions of control values extracted from [39].

## Data Availability

All the data generated during the research are reported in the manuscript.
